# Bacterial cell envelope components of gut commensals: effects in host-microbe interaction

**DOI:** 10.1080/07853890.2026.2618429

**Published:** 2026-01-30

**Authors:** Thaís Vilela Rodrigues, Luís Lima de Jesus, Philippe Langella, Siomar de Castro Soares, Gwénaël Jan, Vasco Ariston de Carvalho Azevedo, Jean-Marc Chatel

**Affiliations:** a Institute of Biological Sciences, Federal University of Minas Gerais, Belo Horizonte, Brazil; b MICALIS, Université Paris-Saclay, INRAE (National Institute for Agriculture, Food, and Environment), AgroParisTech, Jouy-en-Josas, France; c Institute of Biological and Natural Sciences, Federal University of Triângulo Mineiro, Uberaba, Brazil; d Institut Agro, STLO, INRAE, Rennes, France

**Keywords:** Effector molecules, surface bacterial components, gut microbiota, host interaction, immunology

## Abstract

The bacterial cell envelope is a complex structure composed of proteins, lipids, and other molecules that form the physical boundary between bacteria and the environment. In the gut microbiome, commensal bacteria play a fundamental role in maintaining intestinal homeostasis and modulating host physiology. Due to their strategic location, bacterial cell envelope components interact directly with the host immune system, intestinal cells, mucus, and other gut structures, leading to diverse biological effects. Even though beneficial bacteria produce effector molecules, the functional diversity of these molecules, especially among gut bacteria, remains largely unknown. This review compiles current knowledge on the structure and function of bacterial cell envelope components, with a specific focus on gut microbiome commensals and their role in host interactions. It highlights key effector molecules, their immunological recognizers, and the downstream mechanisms involved in such microbial-host interactions, in addition to stating important knowledge gaps. Identifying and characterizing these molecular interactions is a crucial step toward developing novel biotherapeutic targets, particularly for inflammatory bowel diseases and other gut-related disorders. By reviewing current findings and outlining key research deficits, this review deepens our understanding of how bacterial cell envelope components shape host-microbe interactions.

## Introduction

The human gastrointestinal tract harbors a vast microbial community known as the gut microbiome, which includes bacteria, archaea, viruses, and fungi that coexist symbiotically and play a crucial role in host homeostasis and metabolism [1]. Among these microorganisms, commensal bacteria are the most abundant group and establish mutualistic relationships that benefit the host without causing harm, contributing to intestinal homeostasis and overall health [2]. These commensal bacteria are essential for key physiological processes, including nutrient metabolism, immune modulation, and protection against pathogens [3].

The colon harbors the highest density of bacteria in the human body, accounting for approximately 70% of the body’s total microbiota. The predominant bacterial phyla include Bacillota (formerly Firmicutes) and Bacteroidetes, followed by Actinobacteria, Proteobacteria, Fusobacteria, and Verrucomicrobia [4]. Key genera, such as *Akkermansia*, *Faecalibacterium*, *Bifidobacterium*, *Bacteroides,* and *Roseburia,* show potential as next-generation probiotics (NGP) due to their critical roles in health and disease prevention [5]. Dysbiosis, or microbial imbalance, is a condition characterized by a disrupted gut microbiota composition, leading to abnormal host-microbiome interactions. This disruption compromises the mucosal barrier, enables systemic microbial dissemination, and heightens susceptibility to infections and immune dysregulation. These disruptions are linked to diseases such as inflammatory bowel disease (IBD), diabetes, and cancer [1,6].

The human gut microbiota constitutes a highly dynamic ecosystem, where commensal bacteria constantly adapt to a wide array of changing conditions. Factors such as host anatomical features, immune responses, age, diet, the use of antibiotics or other medications, exposure to environmental agents, and microbe–microbe interactions all shape the composition and function of this community [4,7]. Within this context, certain commensal bacteria possess the capacity to adopt pathogenic characteristics and are referred to as pathobionts [8–10]. Under a state of eubiosis, characterized by a balanced microbiota, these pathobionts are typically restrained from causing harm by the collective microbial community and host defenses [11,12]. However, dysbiotic environments, where the normal microbial balance is disrupted, support the booming of pathobionts, contributing to the loss of intestinal homeostasis and the development of diseases such as inflammatory bowel disease and metabolic disorders [13–15]. Examples of such pathobionts include adherent-invasive *Escherichia coli* (AIEC) [16], *Clostridioides difficile* [17] and *Helicobacter pylori [18]*, all of which are capable of long-term colonization in the gut without necessarily inducing symptoms, but can cause disease when host or microbiota equilibrium is disrupted [19].

Host-microbiome communication involves various microbial molecules, including metabolites like short-chain fatty acids, secreted proteins, peptides, and bacterial cell components [20,21]. Large-scale studies, including the Human Microbiome Project (HMP) and enterotype studies [22–24], have significantly advanced our understanding of microbial diversity [25]. In spite of successful efforts to characterize microbiota composition, it remains imperative to identify the key molecules influencing host-microbiota interactions, as well as their immunogenic and beneficial properties. The mechanisms by which effector molecules mediate host interactions are relatively well-characterized in model organisms, such as *Lactobacillus spp.* and *Bifidobacterium spp* [26–28]. However, considering the broad diversity of gut microbiota species, much less is known about other gut commensals’ molecular components and their interchange pathways with the host [29–31].

Bacterial cell envelope components are the focus of this review, since they are strategically positioned at the interface between bacteria and the host. These molecules serve as the first point of contact, playing fundamental roles in resilience and growth, immune recognition, adhesion, colonization of the intestinal mucosa, and cellular signaling [20,32]. They act as crucial mediators of localized host-microbiome recognition and immune modulation by interacting with pattern recognition receptors (PRRs) on epithelial and immune cells [33].

## The bacterial cell envelope

**Figure 1.**
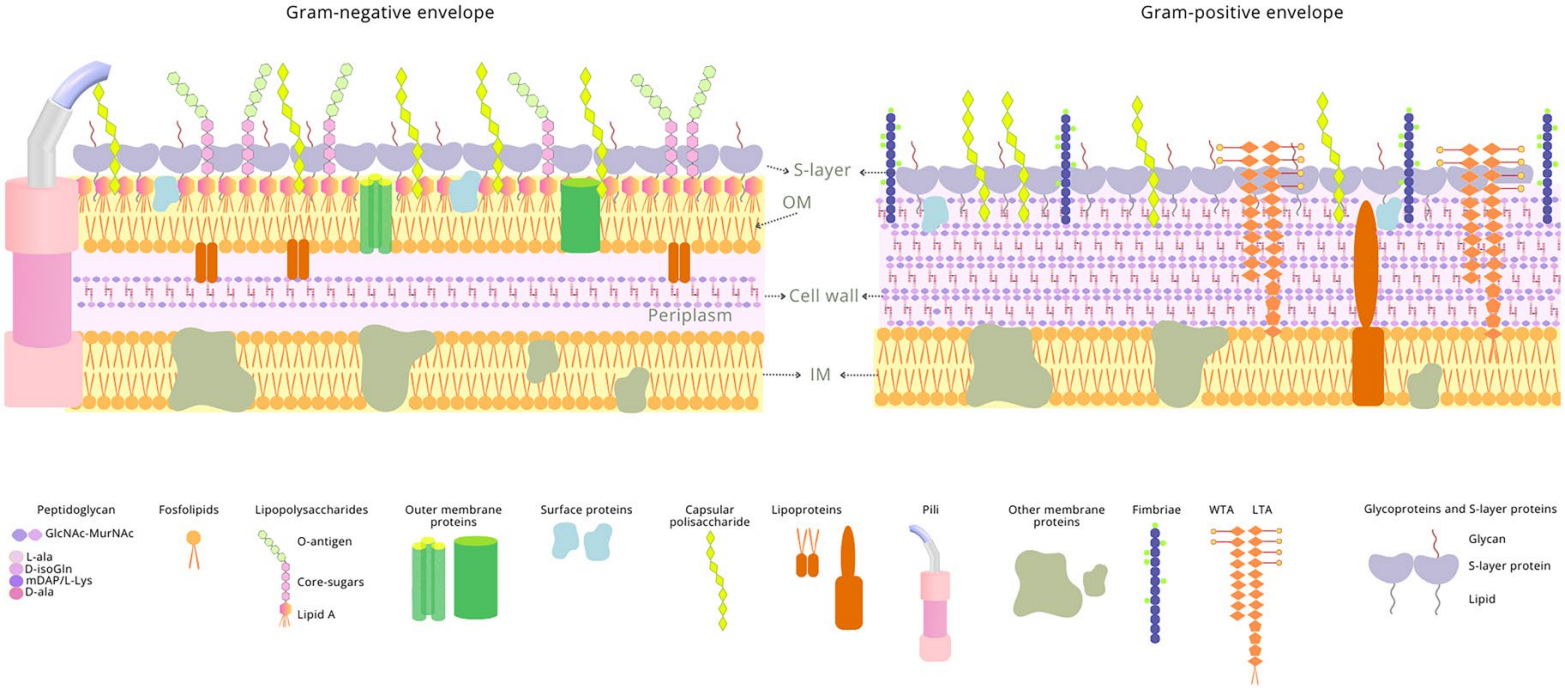
Structural components of Gram-positive and Gram-negative bacterial cell envelopes. The figure illustrates, on the left, the Gram-negative envelope having exclusive components such as the OM with associated LPS, the periplasm, and a thin PG layer. On the right, the envelope of Gram-positive bacteria evidences its thicker PG cell wall and other components, such as TA, fimbriae, and CPS. Below the cell envelope representations, a copy of each one of the elements and their respective names is shown).

The bacterial cell envelope is a complex, multi-layered structure that provides protection, regulates the diffusion of nutrients, and mediates bacterial interactions with the environment. Traditionally, bacteria are classified as Gram-positive or Gram-negative based on their envelope characteristics (Figure 1): Gram-positive bacteria have a thick peptidoglycan layer. In contrast, Gram-negative bacteria possess both a thin peptidoglycan layer and an outer membrane (OM) [34]. Despite the widespread use of this dichotomy, recent studies have revealed diverse cell envelope organizations that deviate from these categories, including additional layers such as intra-cytoplasmic membranes and S-layers, having variable composition of proteins, lipids, and other components yet to be further explored [35].

The structural diversity of bacterial cell envelopes reflects their adaptability to different ecological niches and environmental pressures, including fluctuations in temperature, osmolarity, and biochemical conditions [36]. Beyond these structural functions, the cell envelope, being the primary interface for bacterial interactions with the host and other microbes, has a central role in adhesion, immune recognition, and colonization [20,37]. Such roles are accomplished by a broad range of molecules, whose composition and structure vary markedly among commensal species and even strains, reflecting their adaptation to distinct ecological niches and host environments. This section provides an overview of the key components and organization of the cell envelope, focusing on features relevant to host-commensal interactions.

### Cell envelope organization and components

The bacterial cell envelope consists of multiple layers, including the inner membrane (IM), peptidoglycan (PG) layer, and, in Gram-negative bacteria, the OM. Each of these components has unique structural and functional roles that contribute to bacterial survival and host interactions.

#### Inner membrane

The IM, also known as the cytoplasmic membrane, is a phospholipid bilayer embedded with transmembrane proteins and lipoproteins. It facilitates envelope biogenesis, metabolism, protein secretion, and nutrient transport, serving as the foundation for cell envelope assembly [35,38].

#### Outer membrane

Exclusive to Gram-negative bacteria, the OM is an asymmetric lipid bilayer with lipopolysaccharides (LPS) in its outer leaflet and phospholipids in the inner leaflet. This structure forms a robust permeability barrier, incorporating LPS, lipoproteins, and porins to regulate nutrient diffusion and environmental sensing [38].

#### Peptidoglycan

The peptidoglycan layer is a mesh-like structure composed of glycan chains cross-linked by peptides, providing shape and rigidity to the bacterial cell. In Gram-negative bacteria, it is thin and located within the periplasmic space, while in Gram-positive bacteria, it is much thicker (10–30 times thicker) and cross-linked to provide additional stability. This layer is critical for bacterial growth, septation, and survival under environmental stress [36,39].

#### Teichoic acids (TAs)

Teichoic acids are anionic polymers unique to Gram-positive bacteria, where they are either covalently attached to the peptidoglycan (wall teichoic acids, WTAs) or anchored to the membrane (lipoteichoic acids, LTAs). These molecules contribute to structural integrity, ion transport, and interactions with host tissues, making them key components in adhesion and immune modulation [40].

#### Surface polysaccharides (PSA)

Polysaccharide layers, including capsular polysaccharides (CPS) and exopolysaccharides (EPS), form the outermost interface of bacterial cells. These carbohydrate structures are critical for adhesion, biofilm formation, and immune evasion. Their structural diversity allows bacteria to adapt to varying environments and mediate host interactions [40].

#### Pili and fimbriae

Pili and fimbriae are proteinaceous appendages that facilitate bacterial adhesion to host cells and mediate aggregation with other bacteria. These structures contribute to bacterial colonization and biofilm formation, playing a crucial role in the persistence and resilience of commensal bacteria within host-associated environments [41].

#### S-Layers

S-layers are proteinaceous two-dimensional arrays that coat the cell surface in some Gram-positive and Gram-negative bacteria. These layers provide structural support, mediate adhesion, and protect against environmental stress. While not present in all bacteria, S-layers are significant in certain commensal species, where they facilitate host colonization and immune interactions [42,43].

### Host receptors for envelope molecule identification

The intestinal lumen harbors trillions of bacterial cells that interact constantly with the host intestinal mucosa, triggering both innate and adaptive immune responses that can vary based on the microbiota community [44]. To maintain immune balance, the host must accurately detect and respond to microbial signals which are recognized by PRRs such as Toll-like receptors (TLRs), NOD-like receptors (NLRs), and C-type lectin receptors (CLRs) [45]. These receptors detect conserved microbial molecular patterns, known as microbe-associated molecular patterns (MAMPs), including PG, proteins, LTA, and LPS.

PRR activation regulates both immunity and intestinal barrier integrity, collectively shaping responses that promote tolerance to commensals while remaining poised to counteract pathogens [46,47]. Although these receptors recognize conserved motifs, subtle structural variations in microbial components and host factors determine whether the outcome is pro-inflammatory or anti-inflammatory responses [20] Disruptions in PRR signaling can shift this balance, leading to uncontrolled immune activation. Dysregulated PRR activity, often influenced by alterations in gut microbiota composition, is implicated in chronic inflammatory conditions such as IBD [45,48].

Below, we provide an overview of the primary receptors involved in the bacteria-host interactions.

#### Toll-like receptors (TLRs)

TLRs are transmembrane immune sensors that distinguish commensals from pathogens by recognizing specific cell envelope components and modulating host responses [49]. TLRs are localized on the cell surface or within intracellular compartments, within IECs, and in innate immune cells. They comprise transmembrane proteins with an extracellular leucine-rich repeat (LRR) domain responsible for ligand binding and an intracellular Toll/interleukin-1 receptor (TIR) domain, which mediates downstream signaling [50]. TLR may recognize various MAMPs, including lipoproteins, peptidoglycan, LTA, or glycoproteins. Upon activation, TLRs relay signals to the cell’s interior *via* various adaptor molecules, including Myeloid Differentiation Primary-Response Protein 88 (MyD88). This activation of downstream intracellular signaling pathways triggers immune responses, producing inflammatory markers such as cytokines, anti-microbial peptides (AMPs), and tight junction proteins [51].

Despite continuous exposure to TLR ligands in the gut lumen, IECs express low baseline levels of TLRs. This controlled expression helps maintain tolerance to commensal bacteria and prevents unnecessary inflammation. However, TLR expressions are upregulated during pathogenic infections, with TLR2, TLR4, TLR5, and TLR9 being particularly responsive [52]. Therefore, TLR signaling must be carefully regulated in the gut epithelium. In response to TLR activation, proteins such as Toll-interacting protein (Tollip), A20, and SIGIRR (Single immunoglobulin interleukin-1 receptor-related molecule) are upregulated and act as negative regulators, preventing prolonged or excessive TLR activation and mitigating the risk of inflammation in response to commensal bacteria [53–55].

Disruption of the equilibrium between TLR signaling and gut microbiota can lead to immune dysregulation and the development of various diseases. For example, the expression of TLR4 and TLR2 has been demonstrated to be increased in the intestinal mucosa of patients with ulcerative colitis (UC), which leads to the activation of the TLR/Nuclear factor kappa B (NF-κB) signaling pathway, with the subsequent release of pro-inflammatory cytokines. This increased activation is also associated with changes in the gut microbiota, including an overrepresentation of pathogenic bacteria such as *Enterobacteriaceae* and *Streptococcus*, as well as alterations in the load of beneficial species like *Faecalibacterium* [56,57].

Although TLR2 is broadly described as a sensor of Gram-positive cell wall components, its direct role in PG recognition remains controversial. Some studies indicate that TLR2 primarily senses lipoproteins or LTAs rather than PG itself, whereas others propose that TLR2 can mediate PG internalization and cooperate with NOD2-dependent signaling to modulate immune activation and epithelial barrier function. Such findings suggest that TLR2 involvement in PG sensing may be indirect and context-dependent, influenced by cellular type and microbial source [58–60].

#### NOD-like receptors (NLRs)

NLRs are located in the cytoplasm of various cell types, including IEC and immune cells such as macrophages, lymphocytes, and dendritic cells [61]. NLRs possess an N-terminal interaction domain, which may be a CARD (Caspase Recruitment Domain) or PYRIN domain. This N-terminal interaction domain is followed by a central NOD domain and a C-terminal LRR domain [62].

NODs contribute to the activation of key immune pathways, such as NF-κB and MAPK (Mitogen-Activated Protein Kinase). These receptors cooperate with TLR signaling, in which NOD1 signaling in dendritic cells promotes their maturation into a tolerogenic state, which produces cytokines such as interleukin-10 (IL10). IL10 facilitates the differentiation of regulatory T cells (Tregs), which are crucial for preventing excessive immune reactions against commensals [63,64].

NLRs maintain intestinal barrier integrity and modulate host-microbiota interface through signaling pathways involving caspase-1, RIPK2 (Receptor-interacting protein kinase 2), and NF-κB signaling [65]. Specific NLRs have distinct roles in maintaining gut homeostasis. For instance, NLRP6 promotes *Akkermansia muciniphila* colonization and protects against colitis by enhancing IL18 expression, which is essential for epithelial repair and mucosal integrity [66]. Similarly, NLRP3 regulates IL1β and IL18 production to facilitate epithelial proliferation and immune modulation [65,67]. Dysregulation of NOD2 and NLRP3, has been closely linked to intestinal inflammation, interfering with microbiota composition and epithelial barrier function [68]. NOD2 deficiency was associated with impaired Paneth cell bactericidal activity and increased intestinal inflammation, driven by reduced antimicrobial peptide production and dysregulated commensal bacteria [69]. Additionally, NLRP12 supports gut homeostasis by suppressing excessive inflammatory cytokines, including IL6 and IL1β, and maintaining protective commensal strains [70].

NLRs play another important role in gut homeostasis by detecting bacterial membrane vesicles (BMVs) produced by commensal and probiotic bacteria. Through the activation of NOD1 and NOD2 receptors, these vesicles modulate immune responses by inducing IL10 production, contributing to maintaining a balanced immune environment in the gut [71].

#### C-type lectin receptors (CLRs)

CLRs form a diverse superfamily of transmembrane or soluble PRRs primarily expressed by myeloid cells, such as dendritic cells and macrophages [72]. This family includes receptors like DC-SIGN (dendritic cell-specific ICAM-3-grabbing non-integrin), Dectin-1, Dectin-2, Dectin-3, and macrophage-inducible C-type lectin (Mincle). CLRs contain one or more C-type lectin-like domains (CTLDs) that recognize carbohydrate structures, including β-glucans, mannose-rich glycans, and fucose, which are found on the surface of pathogens, as well as damage-associated molecular patterns (DAMPs) from self-antigens [73,74].

CLRs are well known for their role in recognizing PAMPs, but their interactions with commensal bacteria are still limited [31,75]. Among their properties, the antifungal activity is mediated by Dectin-1, which recognizes β-glucans in *Candida albicans*, while Dectin-2 detects fungal α-mannans. These protective interactions are particularly important during dysbiosis, helping to prevent the overgrowth of harmful species and maintain immune homeostasis [73].

Mincle and Dectin-3 are involved in responses to both bacterial and fungal ligands. For example, Mincle recognizes bacterial glycolipids from *Listeria monocytogenes* and *Escherichia coli*, both associated with inflammatory diseases such as Crohn’s disease [76]. Similarly, Dectin-3 plays a role in antifungal immunity and colitis protection, highlighting its potential contribution to gut immune regulation [76].

The recognition of microbial molecules by host receptors such as CLRs, TLRs, and NLRs demonstrated the complexity of host-microbe interactions in the gut. Despite the progress made in the relations of those key recognition molecules towards gut commensals, there is still a long way to go to determine what the molecules involved in this recognition are, especially when they share similarities with pathogens [65,77]. In the next section, we delve into the specific components of the bacterial cell envelope, exploring their structural roles and the mechanisms by which they influence host physiology and immunity.

### Bacterial cell envelope molecules and host effects

Bacterial surface molecules play a crucial role in host-microbe communication, as they constitute the initial point of contact between the host and the microbe. In this context, unlike pathogens [78] and well-characterized probiotic species [21,79,80], the functional properties of the cell envelope structures of commensal bacteria remain relatively underexplored in the scientific literature [32,81,82].

**Figure 2.**
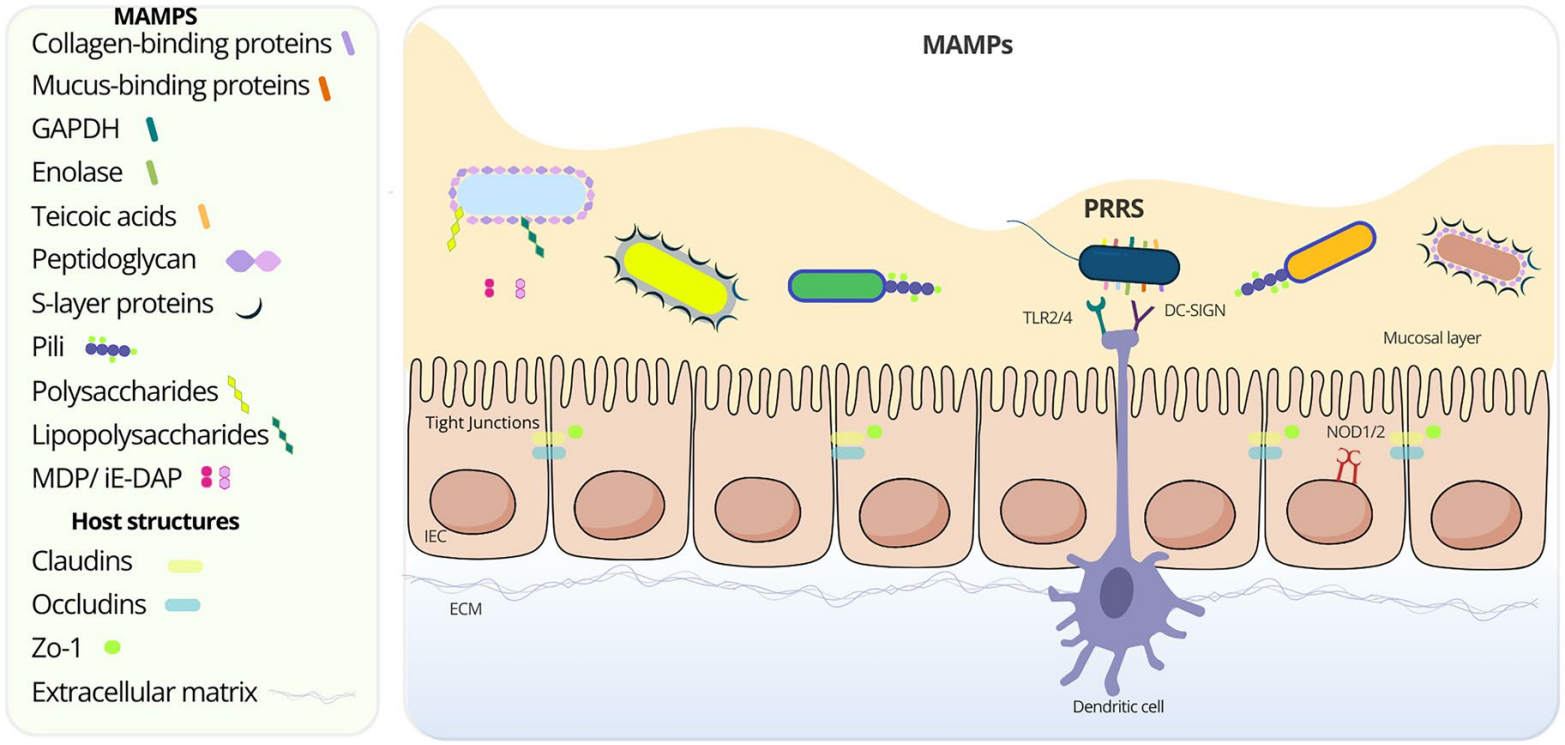
MAMPs from gut microbiota in association with the host intestinal mucosa receptors. This figure depicts the interaction of bacterial MAMPs with the intestinal mucosa. It illustrates various bacterial surface molecules, including collagen-binding proteins, mucus-binding proteins, GAPDH, enolase, teichoic acids, peptidoglycan, S-layer proteins, pili, polysaccharides, lipopolysaccharides, and MDP/iE-DAP. The intestinal epithelial layer consists of IECs, and tight junctions, which include Claudins, Occludins, and Zona occludens-1 (Zo-1). A dendritic cell extends projections below the epithelium. The ECM is shown beneath the epithelium, and the mucosal layer is represented at the apical side of the epithelial barrier in yellow. Host PRRs, including Toll-like receptors (TLR2/4), DC-SIGN, and NOD1/2, are displayed.

**Figure 3.**
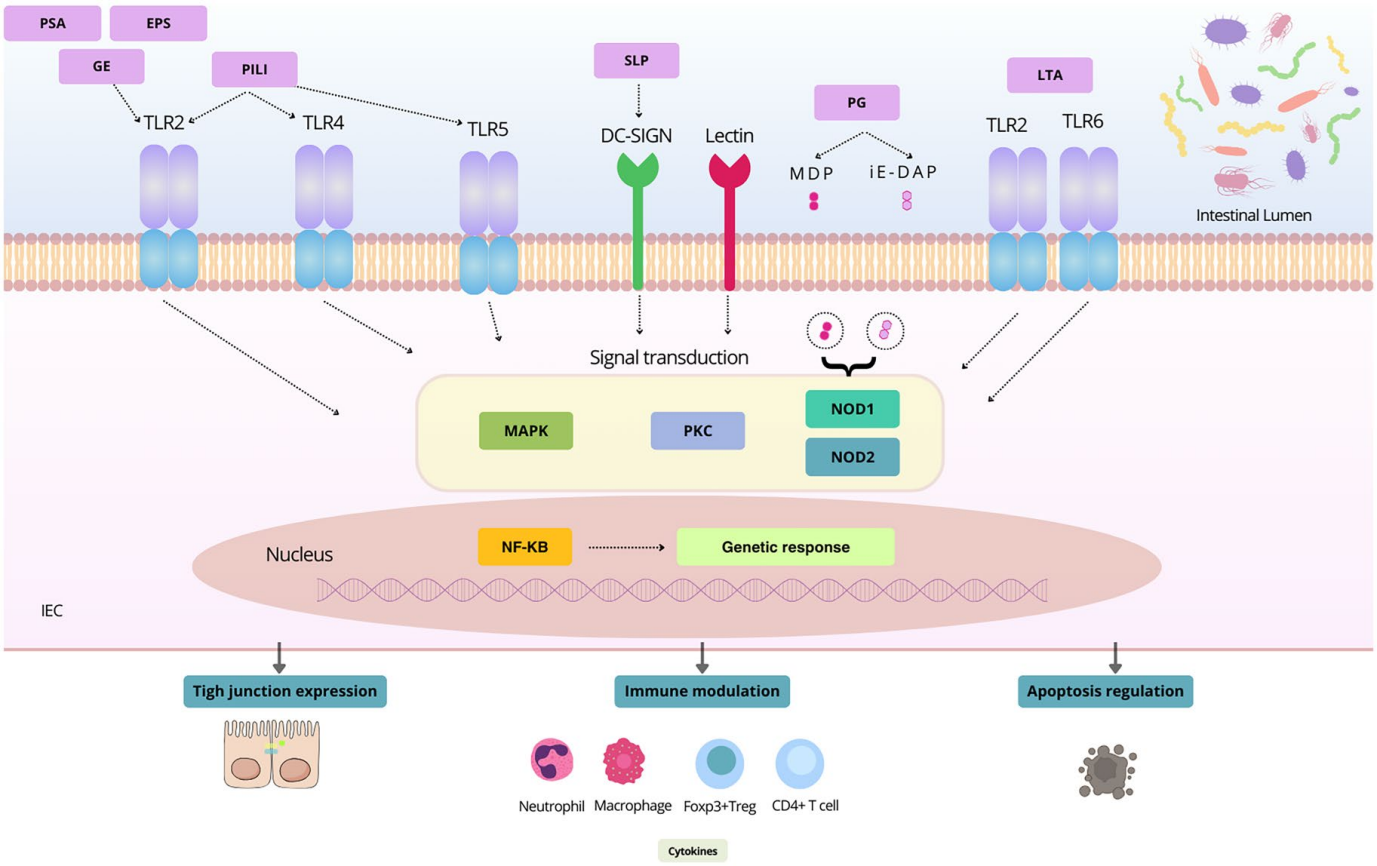
Immunological signalling pathways activated by MAMPs in the intestinal mucosa. This image illustrates an IEC highlighting the main immunological pathways activated by bacterial MAMPs. Various bacterial components, including PSA, EPS, glycolytic enzymes, pili, PG, S-layer protein (SLPs), and LTA, interact with key PRRs such as TLR2, TLR4, TLR5, TLR6, as well as lectins and DC-SIGN. Upon recognition, these receptors initiate signal transduction pathways like MAPK, PKC, and NOD1/2, which activate transcription factors such as NF-κB. This leads to genetic responses influencing tight junction expression, immune modulation (impacting neutrophils, macrophages, Foxp3+ Tregs, and CD4+ T cells), cytokine production, and apoptosis regulation.

In this section, we focus exclusively on specific cell envelope molecules that play a defined role in host-commensal interactions (Figure 2, Table 1). By narrowing our scope to molecules with a known mechanism of action in host modulation, we aim to highlight their effects on immunity (Figure 3), gut barrier integrity, and homeostasis. This approach does not aim to discuss all bacteria with probiotic or commensal properties exhaustively but rather to provide a molecular perspective on the mediators involved in these interactions and their specific contributions to the host.

**Table 1.** Representative examples of gut commensal cell envelope molecules and their characterized or proposed host receptors.

Bacterial specie / strain	Cell envelope molecule	Host receptor (s)	Immunological/ physiological effect	Reference (s)
*Akkermansia muciniphila*	Peptidoglycan fragments	NOD1, NOD2	Activation of innate immune responses	[83]
*Bacteroides fragilis*	PSA (ZPS)	TLR2	Induction of Treg cells, anti-inflammatory response	[84–88]
*Ruminococcus gnavus*	EPS	TLR4	Modulates IL6 and IL10 *via* NF-κB pathway	[89]
*Bacteroides fragilis*	LPS	TLR4	Modulates immune responses, protects intestinal barrier	[90,91]
*Bacteroides vulgatus mpk*	LPS	TLR4	Modulates immune responses	[92]
*Lactobacillus rhamnosus GG*	LTA	TLR2 (with TLR6, CD14)	Modulates immune responses	[93,94]
*Bifidobacterium lactis/bifidum/longum*	Enolase (ENO), GAPDH	Plasminogen	Adhesion to mucosal surfaces	[95]
*Lactobacillus plantarum LM3*	Alpha-enolase	Type I collagen	Adhesion to mucosal surfaces	[96]
*Lactobacillus plantarum LA 318*	GAPDH	Mucin	Adhesion to mucin, blocks pathogen binding	[97]
*Lactobacillus johnsonii MG*	GAPDH	JAM-2	Restores tight junctions in IECs	[98]
*Lactobacillus johnsonii NCC533*	EF-Tu, GroEL	IECs, mucin, CD14	Modulates immune responses, adhesion to mucosal surfaces	[99,100]
*Lactobacillus rhamnosus GG (LGG)*	Pili (SpaCBA)	DC-SIGN, TLR4	Adhesion to mucosal surfaces, modulates immune responses	[101,102]
*Akkermansia muciniphila*	Pili-like (Amuc_1100 protein)	TLR2	Modulates immune responses	[103]
*Escherichia coli Nissle 1917*	F1C fimbriae	TLR4	Adhesion tomucosal surfaces, strengthens intestinal barrier	[104,105]
*Lactobacillus reuteri*	MapA, CBP (collagen-binding)	Mucus, Type I collagen	Adhesion to mucus and collagen	[106,107]
*Lactobacillus plantarum*	CBP	Type I collagen	Adhesion to collagen, protects against pathogens	[106]
*Lactobacillus acidophilus*	SlpA	DC-SIGN, SIGNR3	Modulates immune responses	[108–110]
*Lactobacillus brevis*	SLP	Mincle (CLR)	Modulates immune responses	[111]
*Bifidobacterium adolescentis BB-119*	Surface proteins (36 kDa, 52 kDa)	Type V collagen	Adhesion to collagen	[112]
*Lactobacillus acidophilus NCFM*	MUB	IECs, mucin	Adhesion to mucosal surfaces	[113]
*Lactobacillus reuteri*	MUB, CmbA (mucus-binding proteins)	CLR	Modulates immune responses	[114]
*Bifidobacterium bifidum ATCC 15696*	Sialidase SiaBb2, Tal	Mucin	Adhesion to mucin, mucosal colonization	[28,115]
*Akkermansia muciniphila*	Mucin-binding proteins	O-glycans	Adhesion to mucin, colonization	[116]

Effects are summarized from experimental studies where available. For many commensals, precise and universal molecule–receptor pairing remains strain- or context-specific and, in some cases, is proposed based on experimental evidence rather than fully confirmed.

#### Peptidoglycan in host interaction

PG is one of the most abundant components in the bacterial cell wall. It contributes to gut homeostasis and immune processes, including gut-brain axis signaling and stress responses [117]. Microbiota-derived PG was found to be systemically correlated with innate immune response regulation. PG, as well as its constitutive small molecules: Myramyl dipeptide (MDP), N-Acetylglucosamine (NAG), and γ-D-glutamyl-meso-Diaminopimelic acid (iE-DAP), induce signals *via* several innate immune receptors [118]. PG can be recognized on the bacterial surface, or its fragmented molecules can disseminate through the host intestinal barrier, leading to a variable range of responses through its recognition, from physiologic to pathogenicity effects [119].

The role of TLR2 in recognizing PG remains a topic of debate. While some studies suggest that PG is a ligand for TLR2, others indicates that lipoproteins or LTA, which co-purify with PG, are the actual TLR2 ligands [59]. Supporting this, further purification of PG has been shown to diminish TLR2 activation, indicating that PG alone may not be sufficient to trigger TLR2 signaling [120]. However, soluble PG from penicillin-treated bacteria has been reported to activate TLR2 and induce TNF production in macrophages [121,122]. Additionally, muramyl tripeptides and muramyl tetrapeptides have been found to bind TLR2 *in vitro*, and highly purified PG from various Gram-positive and Gram-negative bacteria can activate human monocyte MonoMac6 cells [122].

Adding complexity, a study on *Bacillus anthracis* demonstrated that PG-induced pro-inflammatory cytokine production required its internalization and degradation, suggesting an additional regulatory mechanism [118]. These conflicting findings likely stem from variations in PG structure across bacterial species and differences in purification methods. Given that TLR2 plays a key role in recognizing commensals and promoting immune tolerance, it is possible that structural variations in PG influence how commensals modulate host immunity [119,123]. Unlike pathogenic PG, which may trigger inflammatory responses, PG from commensals could contribute to gut homeostasis by fine-tuning TLR2 activation. Further studies are needed to explore whether distinct PG modifications in commensal bacteria influence TLR2 signaling and support immune balance [118,124].

Contrastingly with TLR2, the role of NOD1 and NOD2 in sensing PG is well established. These cytoplasmic receptors recognize specific PG fragments to trigger immune responses [118]. MDP has been well-documented as a minimal immunogenic component of PG. The NOD2 ligand is MDP, covalently bound to L-alanyl-L-glutamate, the first two residues of the PG peptide crosslinker [125]. Interestingly, phosphorylation of muramyl peptides by N-acetylglucosamine kinase (NAGK) at the hydroxyl group of its C6 position, yielding 6-O-phospho-MDP, is required for NOD2 activation [126]. This NOD2 ligand is present in PG from both Gram-negative and Gram-positive bacteria. By contrast, the ligand of NOD1, iE-DAP, is found exclusively in Gram-negative bacteria [127]. The binding of the corresponding ligand to the LRR domain leads to self-oligomerization and recruits serine/threonine protein kinase 2 (RIPK2). This interaction activates two distinct signaling cascades: one leading to NF-κB activation *via* phosphorylation of its inhibitor, IκBα, mediated by inhibitory kappa B kinase (IKK), and another activating mitogen-activated protein kinase 7 (MAP3K7, formerly TAK1). Both pathways result in the production of inflammatory cytokines and chemokines, amplifying the immune response [128].

In the context on gut microbiota interactions, PG recognition by PRRs like NOD1 demonstrated enhanced neutrophil activity to defend against pathogens, while healthy microbiota depletion showed increased susceptibility [129]. This activation also leads to macrophage activation and the maturation of dendritic cells to drive an antigen-specific adaptive immune response [62]. In addition, PG fragments from *A. muciniphila* were found to activate both NOD1 and NOD2, despite structural modifications like NAG and N-acetylmuramic acid (MurNAc). This indicates that *A. muciniphila* PG effectively triggers innate immune responses through these receptors, indicating an effect of those molecules in host interactions [83].

As an alternative, PG fragments may be targeted to NOD receptors *via* different transporter-independent mechanisms. Indeed, NOD ligands may be delivered into the cell *via* bacterial secretion systems, pore-forming toxins, or extracellular vesicles (EVs) [130]. EVs are described as molecular cargos and may contain envelope constituents, including PG. Hence, PG from non-invasive commensal bacteria may be delivered to cytosolic NODS through EVs internalized *via* endocytosis [131].

Thus, those studies indicate PG as a key and ample component of host-microbe interactions. Still, much remains unknown about how the host recognizes and interprets its structural variations to drive either pro- or anti-inflammatory responses.

#### Surface polysaccharides (PSA) in host interaction

##### EPS and CPS

Commensal gut bacteria, including *Lactobacilli* [132] and *Bifidobacterium* strains [133], as well as in species such as *Ruminococcus gnavus* [89], and *F. prausnitzii* strain HTF-F [134], are notable producers of PSA, especially EPS. These biopolymers play critical roles in bacterial colonization, gut health, immune modulation, and interactions with host cells, having great importance in probiosis [89,135–137].

Among these*, Bacteroides fragilis* produces the zwitterionic polysaccharide (ZPS) known for its immunomodulatory properties [138]. ZPS carry both positive and negative charges, allowing them to interact uniquely with the host immune system, promoting immunological balance and reducing inflammation [139]. In an *in vivo* study, dendritic cells (DCs) presented surface polysaccharides to CD4+ T cells, leading to Foxp3+ Treg activation, IL10 secretion, and CD39 expression while suppressing pro-inflammatory Th17 and Th2 responses and inducing TH1 cytokines like IFN-γ (Interferon gamma) [84–86]. Similarly, ZPS TP2 from *B. fragilis* strain ZY-312 showed protective effects in a Dinitrobenzene sulfonic acid (DNBS)-induced colitis model and supported the colonization of beneficial gut commensals, including *Faecalibacterium*, and *Ruminococcaceae* [85,140].

In an IBD model, *B. fragilis* PSA demonstrated a protective effect, requiring IL10-producing CD4+ T cells to suppress pro-inflammatory IL17 and TNF production in intestinal immune cells, thereby preventing leukocyte infiltration in colonic tissues [141]. Studies further show that PSA induces cytokine production *via* TLR2-dependent mechanisms [87,88], emphasizing the essential role of PSA-producing commensal bacteria in immune regulation.

Beyond *Bacteroides*, Bifidobacterium-derived EPS has also shown significant immunomodulatory effects. The EPS from *B. longum* BCRC 14634 enhanced macrophage proliferation and IL10 secretion while counteracting LPS-induced TNF-α production and growth inhibition of macrophages. This EPS also demonstrated antimicrobial activity against infectious bacteria. These findings suggest that EPS may function as a mild immune modulator, supporting both immune regulation and antimicrobial defense [142]. In contrast, a mutant for CPS/EPS gene cluster in *B. longum* strain 150-A was efficiently able to bind human intestinal cells. At the same time, the wild-type could not attach to those cells and resisted macrophage internalization, indicating that EPS have a role in bacterial attachment and colonization in the gut [143].

The importance of EPS in immune modulation was further demonstrated in studies with *B. breve* UCC2003. Animals treated with EPS-positive strains did not induce an accumulation of pro-inflammatory immune cells, such as natural killer (NK) cells or neutrophils, in contrast to EPS-deficient strains, which triggered a stronger immune response. Additionally, mice treated with EPS-deficient *B. breve* exhibited higher levels of IL12, IFN-γ, and TNF-α-positive T cells, suggesting that EPS may help *B. breve* evade excessive host immune activation, while its absence provokes a heightened adaptive immune response [144,145]. Regarding other species, the EPS of *B. longum* 35624 was associated with the prevention of exacerbated immune responses triggered by Th17 cells, and the EPS from *B. bifidum* induces the generation of T-reg cells [146].

The gut commensal *Ruminococcus gnavus* also produces different EPS depending on the strain. These molecules can induce different quantities and types of pro- and anti-inflammatory cytokines, including IL6 and IL10, and modulate the NF-κB pathway observed in monocytes. This process was demonstrated to involve signaling *via* immune receptors such as TLR4 [89].

Regarding *Lactobacilli,* their EPS constitutes a key molecule in the formation of biofilm and cross-talk with the host, although duality between pro and anti-inflammatory activity is observed. Different strains of *Lactobacillus* produce EPSs with varying capacities to stimulate pro-inflammatory cytokines such as TNF-α, IL6, and IL12, as well as the anti-inflammatory cytokine IL10 [147]. As an example, the EPS116 from *L. plantarum* NCU116 was associated with improvements in intestinal barrier function by enhancing the expression of Occludin and ZO-1 through the regulation of the STAT3 pathway in both *in vitro* and *in vivo* models, ultimately protecting mice from colitis [148].

Together, these findings highlight the diverse immunomodulatory roles of EPS from different gut commensals, reinforcing their potential to maintain gut homeostasis, modulate host immunity, and even offer therapeutic benefits against inflammatory diseases.

##### LPS

Although LPS is widely studied for its pro-inflammatory properties, it is also involved in antagonistic interactions within the gut microbiota, influencing immune modulation. Commensal bacteria, particularly species from the Bacteroidetes phylum, contribute to nearly 80% of LPS identified in healthy adults [149,150]. This suggests that a delicate balance exists between LPS and mucosal immune mechanisms under normal conditions, allowing tolerance to LPS-containing commensals without excessive inflammation [151].

A key factor in commensal LPS immunomodulation is lipid A modification. Bacteroides species, unlike enterobacterial LPS producers, exhibit hypoacylation and hypophosphorylation of the diglucosamine core, reducing TLR4 activation and LPS toxicity [152]. This structural adaptation, driven by the enzyme LpxF, removes a phosphate group from lipid A, decreasing negative surface charge, which limits AMP binding and dampens immune stimulation [153].

This distinct lipid A modification has been described across several Bacteroides species, influencing their interaction with the immune system. For instance, *B. fragilis* LPS signals through a CD14/MD2-dependent TLR4 pathway but does not activate TLR2, contrary to previous claims. Instead, this structural divergence balances immune responses, promoting gut homeostasis rather than excessive inflammation [90]. In another study, the immunomodulatory effects of Bacteroides LPS are further demonstrated by *B. fragilis* HCK-B3 and *Bacteroides ovatus* ELH-B2, which counteract LPS-induced inflammation by reducing TNF-α and increasing IL10 production, preserving intestinal barrier integrity and restoring the Treg/Th-17 balance, thereby preventing excessive immune activation [91].

Similarly, *Bacteroides vulgatus* mpk LPS has been shown to function as a weak agonist of the MD-2/TLR4 receptor complex, mitigating intestinal inflammation in colitis models. Unlike highly immunostimulatory LPS from proteobacteria, *B. vulgatus* MPK induces semi-mature CD11c + cells in the lamina propria, modulating inflammatory responses without triggering excessive immune activation [92].

Another example is *Bacteroides dorei* LPS, which exhibits significantly reduced TLR4-mediated immune activation compared to *E. coli* LPS. Studies show that *B. dorei* LPS fails to stimulate NF-κB-dependent cytokines (IL10, TNF-α, IL1β, and IL6) in immune cells, whereas *E. coli* LPS induces a strong pro-inflammatory response [154]. These findings reinforce that Bacteroides-derived LPS plays a regulatory role in gut immunity, rather than acting as a classical endotoxin [155].

More broadly, gut commensal LPS contributes to immune crosstalk, regulating host responses. Hennezel et al. demonstrated that LPS from gut-resident microbes antagonizes the TLR4-NF-κB pathway, effectively inhibiting inflammatory cytokine production [149]. In contrast, LPS from pathogenic *E. coli* is highly immunostimulatory, inducing TNF, IL1β, and IL6 *via* TLR4-NF-κB activation [149].

LPS is a key modulator of gut immune balance, but its effects vary widely depending on its structural composition. The presented evidence reinforces the functional divergence of LPS among gut commensals, where modifications in lipid A structure influence bacterial persistence, host-microbe interactions, highlighting that microbiome-derived LPS can facilitate host tolerance to gut microbes, preventing excessive immune activation [156]. Notably, elevated LPS levels in the blood of IBD patients have been observed, but whether this is due to gut barrier dysfunction or other processes remains unclear [157,158]^.^ Understanding these diverse LPS functions is essential for unraveling how gut microbiota shape immune homeostasis and inflammation regulation.

#### Lipoteichoic acids (LTA) in host interaction

Among the earliest studied MAMPs involved in host-microbe crosstalk is LTA, a major structural component of the Gram-positive bacterial cell wall. Known for its role in inducing inflammatory responses, LTA binds to TLR2 and forms a heterodimer with TLR6, CD14, and CD36 (also referred to as GP4), which function as co-receptors [159].

Despite its immunostimulatory nature, LTA from commensal bacteria plays an important role in immune tolerance. TLR2 activation by commensal LTA induces antimicrobial activity in skin mast cells, promoting host defense mechanisms at epithelial barriers [160]. Considering the elevated population of Gram-positive bacteria harboring LTA in the intestinal content, human IECs have adapted as LTA-unresponsive cells, which allows tolerance towards these MAMP, thus avoiding excessive inflammatory response [161]. This tolerance mechanism includes the downregulation of TLR2 co-receptors and the upregulation of Tollip, which inhibits TLR2 signaling. However, macrophages remain highly responsive to LTA, where TLR2 activation plays a key role in immune regulation [162].

The effects of LTA on inflammation vary among commensal species, with different Lactobacillus strains showing contrasting immunomodulatory properties. In a colitis model, *L. rhamnosus* GG worsened dextran sulfate sodium (DSS)-induced colitis in mice. At the same time, its *dltD* mutant disrupted D-alanylation of LTA, hampering LTA biosynthesis, reducing disease severity, and downregulating TLR2 expression and inflammatory cytokines [93]. Furthermore, LTA from *L. rhamnosus* has been shown to induce both pro- and anti-inflammatory cytokines in DCs and T cells, demonstrating a complex role in immune modulation [94].

Contrastingly, LTA from *L. plantarum* has been shown to suppress excessive pro-inflammatory cytokine production, which is in line with the demonstrated ability of an *L. plantarum* lysate to reduce the severity of DSS-induced colitis in rats [163]. Additionally, a mutant of *L. plantarum* with reduced D-alanine incorporation in TA induced higher IL10 production in peripheral blood mononuclear cells (PBMCs), resulting in greater protection against TNBS (2,4,6-trinitrobenzene sulfonic acid)-induced colitis compared to its wild-type (WT) counterpart [164]. Similarly, LTA from *L. paracasei* was found to ameliorate age-related intestinal permeability (‘leaky gut’) and inflammation in mice [165].

These findings highlight that TA and LTA structure and function vary widely among commensal and probiotic bacteria, influencing their immune-modulatory roles. Some LTA molecules drive inflammation, others support immune regulation and gut homeostasis, emphasizing the complexity of bacterial surface components and the strain-specific effects on host interactions.

#### Surface proteins in host interaction

##### Glycolytic enzymes and moonlighting proteins

Glycolytic enzymes, central to energy metabolism, catalyze the conversion of glucose to pyruvate, generating ATP (Adenosine triphosphate), a fundamental process in living organisms [166,167]. Beyond their well-known metabolic role, these enzymes have been found to perform diverse functions, including transcriptional regulation, apoptosis control, and cell mobility. In gut commensal bacteria, they are also involved in adhesion to host mucosa, revealing an intriguing aspect of their multifunctionality [168].

Glycolytic enzymes in gut commensals, particularly enolase (ENO) and Glycoceraldehyde 3-phosphate dehydrogenase (GAPDH), engage in non-metabolic processes like adhesion and interaction with the host. These ‘moonlighting’ proteins- initially known for their intracellular functions- have been identified on the cell surface of various gut-associated bacteria, drive metabolic processes, and facilitate the colonization of the gut mucosa [169–171]. In addition to glycolytic enzymes, other moonlighting proteins, such as elongation factors (EF-Tu and EF-G), hydrolases, chaperones, synthetases, and mutases, have been associated with host-microbe interactions [172].

In *Bifidobacterium* species (*B. lactis*, *B. bifidum*, and *B. longum),* ENO functions as a surface receptor for human plasminogen, suggesting a potential role in host interaction. Other proteins, such as Bile salt hydrolase and Dna K, respectively, glutamine synthetase and phosphoglycerate mutase, were also linked to plasminogen binding in the genus [95]. Similarly, the alpha-enolase of *L. plantarum* LM3 has been shown to bind type I collagen [96]. Moreover, GAPDH adhesive activity was observed in *L. plantarum* LA 318, binding human colonic mucin, potentially hampering pathogens’ attachment in the gut through a competitive mechanism [97].

Other Lactobacillus species have been found to express moonlighting proteins that contribute to adhesion and gut homeostasis. In *L. johnsonii* MG, GAPDH has been demonstrated to specifically bind to junctional adhesion molecule-2 (JAM-2) in Caco-2 cells, where it restores damaged tight junctions [98]. Additionally, in *L. johnsonii* NCC533, elongation factor EF-Tu was identified on the bacterial surface, where it binds to mucins, functioning as an adhesin-like factor [99]. Another study with the same strain identified heat shock protein GroEL as a surface-associated protein capable of binding to mucins and stimulating IL8 production *via* a CD14-mediated mechanism, suggesting a role in gut homeostasis [100].

Beyond their adhesive properties, those moonlighting proteins in gut commensals, such as *Lactobacillus*, have also been linked to immunomodulatory activities, though further studies are needed to clarify their precise mechanisms [173].

These multifunctional roles of moonlighting proteins in gut microbiota-host interactions, particularly in adhesion to the intestinal mucosa, facilitate bacterial colonization and contribute to gut homeostasis. Given that some moonlighting proteins are shared between commensals and pathogens, understanding their binding mechanisms may provide insights into microbiota stability and competitive exclusion. Nevertheless, the processes by which these proteins are secreted and anchored to the bacterial surface remain to be determined.

#### Pili

Proteinaceous appendages on the bacterial cell surface, including flagella, fimbriae, and pili, are widespread in bacterial communities [32]. Pili are key molecules involved in host-microbiota communication because of their adhesive and immunomodulatory properties. They interact with macrophages, being recognized by TLR2 receptors, also regulating pro- and anti-inflammatory cytokine production. Among the different types of pili, Type IV pili (T4P) is among the most abundant [174]. Genomic analyses of gut microbiota species have revealed that approximately 30–45% of tested bacterial species harbor the necessary genes for T4P production [32].

In human intestinal cell assays, the presence of pili promoted bacterial attachment and was associated with reduced IL8 mRNA expression, indicating an indirect anti-inflammatory effect [175,176]^.^ In the commensal *L. rhamnosus* GG (LGG), pili facilitate attachment to intestinal cells and interactions with the mucus layer. Notably, strains lacking the SpaCBA pili system genetic region show reduced adhesive capacities [101].

Post-translational modifications of pili in Gram-positive bacteria, particularly glycosylation, are processes commonly mediated by sortases. In LGG, glycan modifications in SpaCBA pili have been shown to interact with the DC-SIGN lectin on human dendritic cells. These glycan-associated MAMPs trigger distinct signaling pathways, where mannose residues activate TLRs and promote pro-inflammatory cytokine production, whereas fucose induces IL10 responses while inhibiting IL6 and IL12 secretion [174].

Another study with human fetal ileal organ culture and IECs, demonstrated that the SpaC of *L. rhamnosus* (ATCC 53013) modulates host immune responses by reducing TLR4 expression and downregulating IL1β-induced IL6 secretion, contributing to anti-inflammatory effects in IECs [102].

In Bifidobacterium species, pili have been identified as critical for gut colonization and host interaction. In *B. breve* UCC2003, the *tad* locus (Type IVb tight adherence pili) is conserved across strains and is essential for murine gut colonization [177]. Additionally, TadE, a structural component of Tad pili, was found to induce intestinal epithelial cell proliferation. Although the underlying mechanisms remain unclear, evidence suggests that TLR receptors may be involved in this process [178].

Similarly, in *B. bifidum* PRL2010, pili play an essential role in bacterium-host interactions. Using *L. lactis* as an expression system, pil3PRL2010 pili showed significant adhesion to Caco-2 cells, while pil2PRL2010 did not, demonstrating species- and substrate-specific adhesion properties. Bifidobacterium pili also bind to ECM proteins, such as fibronectin, plasminogen, and laminin, with their immunomodulatory effects varying by species. In *B. bifidum* PRL2010, pili induced TNF-α production while reducing IL10, indicating a role in immune modulation [179].

Pili-like structures in *A. muciniphila* have also been identified, encoded by the surface-exposed Amuc_1100 protein, which induces TLR2 signaling and cytokine production (IL6, IL8, and IL10) in PBMCs, suggesting an immune-regulatory function [103]. Since *A. muciniphila* recognizes N-acetyllactosamine for O-glycan attachment, further research is needed to determine whether pili contribute to this adhesion mechanism [116].

Another example of bacterial appendages is the F1C fimbriae from *Escherichia coli* Nissle 1917, which are a key factor in the adhesiveness to abiotic or biotic environments. This property is crucial in biofilm formation and commensal colonization [104]. The recognition of these molecules by the host cell is mediated by receptors such as TLR4, which respond to fimbriae from both commensal and pathogenic bacteria. In *E. coli*, fimbriae can engage distinct co-receptors and adaptor proteins within lipid rafts, triggering signaling pathways that influence bacterial clearance. In commensals, these interactions often promote beneficial colonization, strengthening the gut barrier and modulating immune responses [105].

#### Collagen-binding proteins (CBP) and S-layer proteins

The stable attachment of bacteria to host tissues is a critical step for gut colonization and immune modulation. Gut commensal bacteria use surface proteins like CBPs and SLPs as adhesive mediators, which play essential roles in their interaction with the host’s intestinal mucosa [106]. The ECM, rich in components such as collagen, laminin, fibronectin, and mucin, provides surfaces for bacterial adhesion. Collagen, particularly type V, is abundant in the intestinal mucosa and facilitates bacterial adherence [180]. CBPs mediate direct interactions with ECM components and, together with SLPs, they have a dual purpose: they favor adhesion to the host’s tissues and protect against pathogens, thus contributing to the maintenance of gut homeostasis [106,181].

Many *Lactobacillus* isolates demonstrate collagen-binding capabilities through various adhesin proteins, including CBP, which has been linked to colonization processes that reduce pathogen attachment [106]. For instance, *L. reuteri* expresses the mucus adhesion-promoting protein (MapA), which binds to mucus, collagen type I, and Caco-2 cells [107]. Similarly, CBPs derived from *L. plantarum* Lp91 have shown strong adhesive capacity and the ability to inhibit *E. coli* O157 attachment to human type I collagen *in vitro* [106].

SLPs have pivotal participation in immune regulation, protecting the intestinal barrier, and inhibiting pathogen colonization. A well-characterized example is *L. acidophilus*, which possesses a complex S-layer composed of major SLPs (SlpA, SlpB, and SlpX). SlpA (S-layer protein A) functions as a commensal ligand that binds to DC-SIGN receptors, promoting a Th2 immune response [108]. *L. acidophilus*, or its purified SlpA, has been shown to protect mice from inflammation and dysbiosis in a murine colitis model. This effect is mediated by the recognition of the SLPa by a C-lectin receptor called SIGNR3 (specific intracellular adhesion molecule-3 grabbing non-integrin homolog-related 3) [109]. *L. acidophilus* NCFM’s SLP has also been shown to reduce the production of IL-1β, TNF-α, and reactive oxygen species in LPS-stimulated macrophages by inhibiting the MAPK and NF-κB signaling pathways [110]. In another study, the SLP isolated from *L. acidophilus* CICC 6074 activates pro-inflammatory pathways, including MAPK and NF-κB. However, PKC inhibition was also observed, suggesting that this effect can be modulated and reveal a potential duality in its immune function [182]. The S-layer from *L. brevis* was described as recognizing the receptor CLR Mincle. The interaction triggers signaling cascades that induce both pro- and anti-inflammatory cytokine responses, particularly IL10 [111].

Beyond immune modulation, SLPs contribute to the maintenance of gut barrier integrity. For instance, *L. acidophilus* NCFM’s SLP has been associated with maintaining gut barrier integrity in a TNF-α-induced inflammatory model using Caco-2 cells. Notably, these protective effects were absent in SIGNR3-deficient mice [110]. Moreover, it enhanced the expression of tight junction proteins ZO-1 and Occludin in Caco-2 cells subjected to TNF-α-induced inflammation, leading to reduced NF-κB pathway activation, decreased IL8 production, and preventing apoptosis [183]. S-layer beneficial properties have also been observed in *L. plantarum.* SLPs enhanced tight junctions, reduced permeability, and increased transepithelial resistance (TEER) in response to enteropathogenic *E. coli* [184]. In *Bifidobacterium*, two surface proteins from *B. adolescentis* BB-119, with molecular masses of 36 kDa and 52 kDa, bind to type V collagen *via* galactose chains, facilitating bacterial adhesion through lectin-like activity [112]. The protective effects of *L. acidophilus* SLPs have also been demonstrated against pathogens such as *Salmonella typhimurium.* In Caco-2 cells, SLP was associated with blocking signaling pathways, such as ERK1/2, JNK, and p38, thereby reducing IL8 secretion and preserving gut barrier integrity [185].

Taken together, collagen-binding proteins and SLP serve as critical mediators of host-microbe interactions, playing essential roles in bacterial adhesion, immune modulation, and gut barrier integrity.

#### Mucus-binding proteins

The intestinal mucosal layer is a protective matrix coating epithelial cells, protecting against pathogens while supporting gut commensals adhering and thriving within the microbiota. Bacterial adherence to mucus involves nonspecific interactions and mucus-binding proteins (MUBs) on the bacterial surface [186]. Though not fully understood, these proteins binding mucosal carbohydrates represent a key mechanism for bacterial adherence [187].

In several *Lactobacillus* species, MUBs facilitate attachment to the mucosal matrix [188]. The MUB protein from *L. acidophilus* NCFM attaches to intestinal cells and mucin [113]. In the commensal *L. reuteri*, mucus adhesins, including MUB and CmbA, exert immunomodulatory effects on human monocyte-derived dendritic cells, hence modulating the production of anti- and pro-inflammatory cytokines IL10, TNF-α, IL1ß, IL6, and IL12. Purified *L. reuteri* MUB binds to CLRs, inducing Th1-polarized immune responses associated with increased IFN-γ production [114,189,190].

In the context of mucus-binding proteins, studies on *B. bifidum* ATCC 15696 have highlighted the role of the extracellular sialidase SiaBb2 in enhancing mucosal adhesion. This enzyme facilitates the utilization of human milk oligosaccharides and mucin-derived carbohydrates, promoting bacterial adhesion to mucosal surfaces, thereby reinforcing host-microbe interactions [28]. Several *B. bifidum* strains demonstrated the capacity to produce a surface enzyme called transaldolase (Tal). The protein demonstrated autoaggregative properties with a binding capacity to mucin, suggesting participation in gut colonization [115]. Moreover, several mucus-binding proteins that are also moonlighting proteins were identified in EVs derived from *B. longum* NCC2705. The proteins were phosphoketolase, GroEL, EF-Tu, phosphoglycerate kinase, and heat shock protein 20 (Hsp20)

Finally, regarding the NGP *A. muciniphila*, proteins involved in binding O-glycans on mucins were identified. Throughout the activity of neuraminidases, those glycans are exposed to the attachment to mucin-binding proteins, an essential mechanism for bacterial colonization [116].

## Concluding remarks

With a focus on gut commensal bacterial structural and functional diversity of bacterial cell envelope components, this review highlights diverse effector molecules emphasizing their central role as mediators in host–microbiota interactions and maintenance of intestinal homeostasis. These molecules, as the primary interface between commensals and the host, critically shape immune and physiological processes.

However, despite significant advances in microbiota research, there is still limited understanding of the specific molecular mechanisms and effector molecules driving host–microbe communication, especially among less-studied commensal taxa. Focusing future research on underexplored genera such as Roseburia and Ruminococcus will likely reveal new factors and pathways relevant for gut health and disease prevention. Moreover, it is increasingly clear that the distinction between commensal and pathogenic behaviour is highly context-dependent. Even subtle variations in MAMPs can differentially modulate host responses, a complexity that future precision microbiome research will need to address [20,191].

The integration of emerging methodologies, such as CRISPR-based functional genomics, spatial metatranscriptomics, cryo-electron microscopy, and targeted mutagenesis, will be key for uncovering the functions and structural diversity of these envelope components, including EVs, which are often covered by such surface molecules. Combined with *in vivo* and *in vitro* models, these advances set the stage for the rational design of NGP with optimized immunomodulatory properties and future clinical applications. Yet, translating these discoveries into effective therapies faces important obstacles: ensuring the stability and viability of biotherapeutic strains, overcoming challenges in production and distribution, navigating regulatory approval, and achieving consistent efficacy across diverse patient populations. Addressing these issues will be essential for realizing the full potential of microbiota-based interventions in precision medicine.

## Data Availability

Data sharing is not applicable to this article as no new data were created or analyzed in this study.
